# In-solution Y-chromosome capture-enrichment on ancient DNA libraries

**DOI:** 10.1186/s12864-018-4945-x

**Published:** 2018-08-14

**Authors:** Diana I. Cruz-Dávalos, María A. Nieves-Colón, Alexandra Sockell, G. David Poznik, Hannes Schroeder, Anne C. Stone, Carlos D. Bustamante, Anna-Sapfo Malaspinas, María C. Ávila-Arcos

**Affiliations:** 1 Institute of Ecology and Evolution, University of Bern, Bern, Switzerland; 2 International Laboratory for Human Genome Research, National Autonomous University of Mexico, Mexico, Mexico; 3 Department of Computational Biology, University of Lausanne, Lausanne, Switzerland; 4 Swiss Institute of Bioinformatics, Lausanne, Switzerland; 5 School of Human Evolution and Social Change, Arizona State University, Tempe, USA; 6 Department of Genetics, Stanford University, Stanford, USA; 7 23andMe, Mountain View, USA; 8 Natural History Museum of Denmark, University of Copenhagen, Copenhagen, Denmark; 9 Faculty of Archaeology, Leiden University, Leiden, Netherlands; 10 Institute of Human Origins, Arizona State University, Tempe, USA; 11 Department of Biomedical Data Science, Stanford University, Stanford, USA

**Keywords:** Ancient DNA, Capture-enrichment, Y chromosome

## Abstract

**Background:**

As most ancient biological samples have low levels of endogenous DNA, it is advantageous to enrich for specific genomic regions prior to sequencing. One approach—in-solution capture-enrichment—retrieves sequences of interest and reduces the fraction of microbial DNA. In this work, we implement a capture-enrichment approach targeting informative regions of the Y chromosome in six human archaeological remains excavated in the Caribbean and dated between 200 and 3000 years BP. We compare the recovery rate of Y-chromosome capture (YCC) alone, whole-genome capture followed by YCC (WGC + YCC) versus non-enriched (pre-capture) libraries.

**Results:**

The six samples show different levels of initial endogenous content, with very low (< 0.05%, 4 samples) or low (0.1–1.54%, 2 samples) percentages of sequenced reads mapping to the human genome. We recover 12–9549 times more targeted unique Y-chromosome sequences after capture, where 0.0–6.2% (WGC + YCC) and 0.0–23.5% (YCC) of the sequence reads were on-target, compared to 0.0–0.00003% pre-capture. In samples with endogenous DNA content greater than 0.1%, we found that WGC followed by YCC (WGC + YCC) yields lower enrichment due to the loss of complexity in consecutive capture experiments, whereas in samples with lower endogenous content, the libraries’ initial low complexity leads to minor proportions of Y-chromosome reads. Finally, increasing recovery of informative sites enabled us to assign Y-chromosome haplogroups to some of the archeological remains and gain insights about their paternal lineages and origins.

**Conclusions:**

We present to our knowledge the first in-solution capture-enrichment method targeting the human Y-chromosome in aDNA sequencing libraries. YCC and WGC + YCC enrichments lead to an increase in the amount of Y-DNA sequences, as compared to libraries not enriched for the Y-chromosome. Our probe design effectively recovers regions of the Y-chromosome bearing phylogenetically informative sites, allowing us to identify paternal lineages with less sequencing than needed for pre-capture libraries. Finally, we recommend considering the endogenous content in the experimental design and avoiding consecutive rounds of capture, as clonality increases considerably with each round.

**Electronic supplementary material:**

The online version of this article (10.1186/s12864-018-4945-x) contains supplementary material, which is available to authorized users.

## Background

Uniparentally inherited markers such as those on the mitochondrial chromosome (mtDNA) and on Y-chromosome DNA (Y-DNA) are widely used to infer the demographic histories of specific human lineages [1]. Although much smaller than the nuclear genome, the inheritance mechanism and lack of recombination make them powerful tools for inferring ancestry and estimating the ages of pedigrees and the times to the most recent common ancestors (TMRCA) of the mtDNAs and Y-DNAs of present-day populations [2]. Analyses of modern and ancient mtDNA and Y lineages have broadened our knowledge of diversification and founder events from human population history [3–6].

Due to the large number of copies in each cell, mtDNA has been at the forefront of ancient DNA research [7]. In contrast, each cell possesses just one copy of the Y chromosome. Thus, when analyzing ancient samples, the probability of retrieving any given portion of Y-chromosome DNA is much lower than for mtDNA. Furthermore, as endogenous DNA is often found highly fragmented and in low quantity, recovering ancient DNA (aDNA) from a pool of endogenous and contaminating environmental DNA is extremely challenging and costly. To overcome these challenges, methods have been developed to increase the endogenous DNA proportion of sequencing libraries. These methods target select genomic regions, such as SNPs, whole chromosomes, or mitochondrial or nuclear genomes [3, 8–12] prior to sequencing. They consequently increase the proportion of genomic regions of interest while reducing sequencing costs.

There are two main types of enrichment methods: solid phase enrichment [13, 14] and in-solution enrichment [8, 11, 15]. Both approaches require DNA or RNA probes to hybridize to the targeted molecules of a DNA library. The resulting complex (probe and target) is retained either by being attached to the array (solid phase) or pulled down with streptavidin-coated beads (in-solution). Finally, the remaining fragments that did not hybridize with the probes, including those from microbial DNA contamination, are washed away. Capture-enrichment approaches have enabled DNA retrieval from samples that initially showed small amounts of endogenous DNA [10, 16, 17]. Consequently, these enrichment methods have positively impacted ancient genomics research by lowering endogenous content requirements, thereby increasing the number of samples that can be employed for genotyping.

Capture-enrichment strategies have been applied to target genome-wide SNP sets and to specific subsets of the genome to study the phylogenetic context of ancient populations. Recent implementations [5, 8, 12], include probes targeting thousands of autosomal and Y SNPs characterized by the Simons Genome Diversity Project [18] and the International Society of Genetic Genealogy (ISOGG, https://isogg.org/). However, due to the provenance of the samples of the ISOGG consortium, ISOGG SNPs are best suited to genotype present-day European haplogroups. Consequently, aDNA enrichment has been applied to study Y-chromosome variation in ancient European and Middle-Eastern individuals, while studies of Africans [19] and Native Americans have been restricted either to direct interrogation of known Y-DNA markers with targeted PCR-based sequencing [20] or to low and medium-depth whole-genome sequencing [19, 21–23].

An important consideration of enrichment designs targeting pre-selected SNPs is the ascertainment bias introduced and the impediment of discovering new variants. An ideal strategy would involve capturing the whole Y chromosome, however its abundance of repetitive sequences makes it less amenable for capture experiments [15]. To overcome these constrains, we made use of a probe design targeting 8.9 megabases (Mb) out of the 10.3 Mb defined by Poznik and colleagues [2]. These 10.3 Mb were initially selected to fall within the non-recombining portion of the Y chromosome, be depleted of repeats, and well suited for genotype calling from short read sequence data [2]. Furthermore, we were interested in assessing whether Y-DNA could be enriched from libraries with very low endogenous content that had been subjected to WGC and for which we also had pre-capture libraries. We thus tested this approach and compared different enrichment strategies, on samples excavated from the Caribbean, a region that poses a particular challenge for DNA preservation. Previous studies on some of these samples failed to obtain enough Y-DNA data to reliably call a haplogroup even after WGC [24]. Consequently, we investigated the parameters affecting the quality and the quantity of the data and, at the same time, described the extent to which the enrichment improved the resolution of the Y-chromosome haplogroup assignment. Our results illustrate the benefits of Y-DNA enrichment experiments for studying the paternal genetic ancestry of ancient human populations.

## Methods

### Samples

We performed 18 capture-enrichment experiments on DNA libraries obtained from the archaeological remains of six individuals excavated from Caribbean contexts. Two samples (STM1 and STM2) belong to seventeenth-Century enslaved males of African origin from Saint Martin (Lesser Antilles) and were previously reported in [24]. The other four (PI174, PI383, PI435, and PI437) were obtained from archaeological remains from the Paso del Indio site (PI) in Puerto Rico. These four dated between 824 and 1039 CE, as described in [25, 26].

### Ancient DNA extraction

DNA from the STM samples was extracted from tooth roots using a silica-based method [27], as described in [24]. Sampling and DNA extractions for PI samples were conducted at the Arizona State University Ancient DNA Laboratory, a Class 10,000 clean-room facility. Teeth were cleaned with a 1% sodium hypochlorite solution, and the outer surfaces of the tooth roots were mechanically removed with a Dremel tool. Teeth were sliced transversely at the cemento-enamel junction using the Dremel. The roots were then covered in aluminum foil and pulverized by blunt force with a hammer, as in [28]. To avoid contamination, additional precautions were taken, including single use of Dremel wheels, bleach decontamination and UV irradiation of tools and the work area before and between uses, as well as full body coverings for all researchers [29]. DNA was extracted following an improved silica-based extraction protocol [16], using 50 mg of pulverized tooth material. Extracts and extraction blanks were quantified with the Qubit 2.0 High Sensitivity assay [30].

### Ancient DNA library preparation

DNA extracted from STM samples was built into 6-bp-indexed double-stranded Illumina libraries, as described in [24], following the protocol in [31]. For PI samples, double-stranded Illumina libraries were prepared following the protocol in [31]. Extraction blanks were also converted into libraries, and an additional negative library control containing only ddH_2_0 was also included. 1:100 dilutions of each library were prepared for quality screening through Real-Time PCR (qPCR) using the Thermo Scientific Dynamo SYBR Green qPCR kit with ROX. Reactions were run in triplicate and prepared in final volumes of 20 μl with the following conditions: 10 μl of 2X Dynamo SYBR Green qPCR Master Mix with 0.3× ROX, 1 μl of primer IS7 (5’-ACACTCTTTCCCTACACGAC-3′) at 10 μM, 1 μl of primer IS8 (5’-GTGACTGGAGTTCAGACGTGT-3′) at 10 μM, 7 μl of ddH_2_0, and 1 μl of library dilution. Reactions were heated to 95 °C for 10 min for initial denaturation, and further denaturations were performed at 95 °C for 15 s and for 40 1-min cycles at 60 °C. A final disassociation stage was added at the end of these cycles: 95 °C for 15 s, 60 °C for 15 s and 95 °C for 15 s. Quantification was performed using an ABI7900HT thermocycler and analyzed with SDS software. After qPCR, all libraries were double-indexed as in [32]. To retain library complexity, four 100 μl indexing reactions were performed per library with the following conditions: 10 μl of *Pfu* Turbo Buffer, 2.50 μl of 10 mM dNTPs, 1.50 μl of 10 mg/ml Bovine Serum Albumin, 2 μl of P5 indexing primer (5’-AATGATACGGCGACCACCGAGATCTACACxxxxxxACACTCTTTCCCTACACGACGCTCTT-3′) at 10,000 nM, 2 μl of P7 indexing primer (5’-CAAGCAGAAGACGGCATACGAGATxxxxxxGTGACTGGAGTTCAGACGTGT-3′) at 10,000 nM, 72 μl of ddH_2_0, 1.00 μl of *Pfu* Turbo enzyme (Agilent), and 9 μl of DNA library. Reactions were heated to 95 °C for 15 min for initial denaturation. Further denaturation, annealing, and elongation were performed at 95 °C for 30 s, at 58 °C for 30 s, and for 10 45-s cycles at 72 °C. Final extension was performed at 72 °C for 10 min and reactions were then kept at 10 °C. All four aliquots of each amplified library were combined, and the library was purified with the Qiagen MinElute PCR purification kit following manufacturer’s instructions with the following modification: the EB buffer was preheated to 65 °C before use, and reactions were eluted in 30 μl. A 1-μl aliquot of each library was used for quantification with the Qubit 2.0 Broad Range assay. Purified libraries were further diluted to a factor of 1:1000 and quantified with the KAPA Library Quantification kit (Kapa Biosystems) following manufacturer’s instructions.

Indexed libraries were amplified a second time to increase the amount of DNA. To retain library complexity, four 100-μl amplification reactions were performed per library. PCR conditions were: 10 μl of 10X Accuprime *Pfx* reaction mix, 3 μl of IS5 primer at 10 μM, 3 μl of IS6 primer at 10 μM, 76 μl of ddH_2_0, 1 μl of Accuprime™ *Pfx* enzyme, and 7 μl of DNA library. Reactions were heated to 95 °C for 2 min for initial denaturation, and further denaturation, annealing, and elongation were performed at 95 °C for 15 s, 60 °C for 30 s, and for 7–13 1-min cycles at 68 °C. Final extension was performed at 68 °C for 5 min and reactions were then kept at 4 °C. All four aliquots of each amplified library were combined, and the library was purified with Qiagen MinElute PCR purification kit as detailed above. 1 μl of each purified and amplified library were used for flourometric quantification. Purified libraries were further diluted to a factor of 1:10,000 and quantified with the KAPA Library Quantification kit (Kapa Biosystems) following the manufacturer’s instructions. 1 μl of each library was used for fragment analysis with the Agilent 2100 Bioanalyzer DNA 1000 chip.

### Whole-genome capture

Whole-Genome Capture was performed on each of the libraries obtained from the six archaeological samples (STM1, STM2, PI174, PI383, PI435, PI437) following published protocols. We used the human whole-genome enrichment kit MYbaits (MYcroarray, Ann Arbor, online version 1.3.8) to capture STM libraries, as reported in [17]. For PI libraries, we implemented the WISC approach [10], starting with 500 ng per library and hybridizing for 66 h. The libraries were PCR amplified for 15–20 cycles.

### Y-chromosome bait design

We used DNA biotinylated probes (baits) from Nimblegen’s SeqCap EZ Choice XL Enrichment Kit for Y capture. Baits were designed using Roche’s NimbleDesign proprietary probe-design algorithm (https://design.nimblegen.com/nimbledesign) to target 10.3 Mb well suited for reliable genotype calling and haplogroup inference [2]. Out of these 10.3 Mb, the software defined 8.9 Mb (7.4 Mb in 17,934 regions plus 100 bp offset), as suitable for probe design, and returned 2.1 million probes 105 bp in length and an average tiling of ~ 25 bp (~ 4.2 bp between adjacent probes). Probes were designed using the hg19/GRCh37 reference sequence for the Y chromosome, most of which is derived from a single European haplogroup. A file containing the regions defined as suitable for probe design is available in Additional file 1.

### Y-chromosome capture (YCC)

We performed Y-chromosome capture-enrichment experiments on both pre-capture and WGC libraries. Libraries were pooled in equal masses. Capture reactions were performed according to NimbleGen SeqCap EZ XL protocol, with the following modifications: due to limited sample availability, the total mass of the pooled libraries was ~ 500 ng rather than the recommended 1.25 μg; hybridization was performed for a total of 65 h (48–72 h recommended); and the adapter-blocking oligonucleotides were IDT xGen blocking oligos. Following capture, libraries were amplified with 6 cycles of PCR, and quality was assessed using the Agilent Bioanalyzer High Sensitivity kit.

### Illumina sequencing

Pre-capture and WGC libraries for the STM samples were sequenced at the National High Throughput DNA Sequencing Centre in Copenhagen, Denmark, on a HiSeq2000 platform using single-end 100-bp runs, as reported in [17, 24]. PI pre-capture and WGC libraries were paired-end sequenced on the NextSeq500 using a High Output 150-cycle kit with paired-end 76-bp reads. All libraries subjected to YCC (i.e., YCC and WGC + YCC libraries from all samples) were sequenced on the NextSeq500 using the High Output 150 cycle kit at Stanford University using paired-end 76-bp mode.

### Sequence data processing and mapping

FASTQ-format reads from the pre-capture and WGC conditions are available for the STM samples through the European Nucleotide Archive, project PRJEB8269, experiment accession numbers ERX682089, ERX682243, ERX682248, and ERX682249 [24]. We processed these reads, as well as the reads generated for this study (YCC and WGC + YCC libraries for the STM and all PI libraries) with the following steps. To trim adapters and low quality bases, we used AdapterRemoval v2 with the default options in single-end mode for STM pre-capture and WGC libraries, and in paired-end mode for all PI and STM YCC libraries [33]. As the yield per experiment is variable and can bias the comparisons, we subsampled 10 times an equal number of reads for each experimental condition and for each individual using seqtk (https://github.com/lh3/seqtk). To determine the total number of sequences to subsample, we selected the lowest number of reads that passed the trimming filters for each individual across experiments (Table 1). The sequences were then aligned to the *Homo sapiens* reference genome build 37 (hg19) using the BWA aligner [34] implemented in PALEOMIX [35], with a mapping quality threshold set at 30. The quality of the aligned bases was rescaled with mapDamage2 [36] to lower the quality of mismatches to the reference sequence that likely derive from DNA damage.

**Table 1:** Samples, methods, total number (total data) and average (down-sampled data) number of reads, and fold-enrichment (down-sampled data)

Endogenous content (pre-capture)			STM1	STM2	PI174	PI383	PI435	PI437
			1.54%	0.12%	0.01%	0.03%	0.04%	0.02%
Site			Saint Martin	Saint Martin	Paso del Indio	Paso del Indio	Paso del Indio	Paso del Indio
Methods	Extraction		Rohland et al., 2007	Rohland et al., 2007	Dabney et al., 2013	Dabney et al., 2013	Dabney et al., 2013	Dabney et al., 2013
	Library building		Meyer and Kircher, 2010	Meyer and Kircher, 2010	Meyer and Kircher, 2010	Meyer and Kircher, 2010	Meyer and Kircher, 2010	Meyer and Kircher, 2010
	WGC		MYbaits (MYcroarray, Ann Arbor)	MYbaits (MYcroarray, Ann Arbor)	WISC (Carpenter et al., 2013)	WISC (Carpenter et al., 2013)	WISC (Carpenter et al., 2013)	WISC (Carpenter et al., 2013)
	YCC		YCC (this study)	YCC (this study)	YCC (this study)	YCC (this study)	YCC (this study)	YCC (this study)
	WGC + YCC		MYbaits + YCC	MYbaits + YCC	WISC + YCC	WISC + YCC	WISC + YCC	WISC + YCC
Total data	Pre-capture	Total reads	34,025,874	14,973,474	9,173,100	2,795,632	4,617,394	9,986,135
		Mapping to chrY	1384	301	4	3	6	6
		On-target	924	197	1	2	3	2
		% of sequenced on-target	0.00%	0.00%	0.00%	0.00%	0.00%	0.00%
		% of chrY on-target	67%	65%	25%	67%	50%	33%
	YCC	Total reads	68,795	41,884	159,728	107,796	41,810	56,157
		Mapping to chrY	16,430	2562	17	27	26	12
		On-target	16,191	2541	17	27	25	12
		% of sequenced on-target	23.54%	6.07%	0.01%	0.03%	0.06%	0.02%
		% of chrY on-target	99%	99%	100%	100%	96%	100%
	WGC	Total reads	14,973,474	29,884,294	10,052,798	10,265,430	12,363,127	9,132,306
		Mapping to chrY	6407	1925	5	8	57	7
		On-target	2930	903	2	6	29	4
		% of sequenced on-target	0.020%	0.003%	0.000%	0.000%	0.000%	0.000%
		% of chrY on-target	46%	47%	40%	75%	51%	57%
	WGC + Y	Total reads	98,540	30,629	17,212,495	14,557,575	10,181,433	11,356,277
		Mapping to chrY	4854	236	37	152	242	47
		On-target	4643	222	37	150	235	45
		% of sequenced on-target	4.71%	0.72%	0.00%	0.00%	0.00%	0.00%
		% of chrY on-target	96%	94%	100%	99%	97%	96%
Down-sampled data	Pre-capture	Total reads	68,795	30,629	159,728	107,796	41,810	56,157
		Mapping to chrY	2.8	0.2	–	0.2	0.1	–
		On-target	2.3	0.2	–	–	0.1	–
		% of sequenced on-target	0.00%	0.00%	0.00%	0.00%	0.00%	0.00%
		% of chrY on-target	82%	100%	–	–	100%	–
	YCC	Total reads	68,795	30,629	159,728	107,796	41,810	56,157
		Mapping to chrY	16,430	1925	17	27	26	12
		On-target	16,191.0	1909.8	17.0	27.0	25.0	12.0
		% of sequenced on-target	23.54%	6.24%	0.01%	0.03%	0.06%	0.02%
		% of chrY on-target	99%	99%	100%	100%	96%	100%
	WGC	Total reads	68,795	30,629	159,728	107,796	41,810	56,157
		Mapping to chrY	38.8	2.7	0.2	0.1	0.2	–
		On-target	16.8	1.5	0.1	0.1	–	–
		% of sequenced on-target	0.02%	0.00%	0.00%	0.00%	0.00%	0.00%
		% of chrY on-target	43%	56%	50%	100%	0%	–
	WGC + Y	Total reads	68,795	30,629	159,728	107,796	41,810	56,157
		Mapping to chrY	4414.0	236.0	17.1	82.5	111.9	25.5
		On-target	4239.5	222.0	17.1	81.3	109.7	24.5
		% of sequenced on-target	6.16%	0.72%	0.01%	0.08%	0.26%	0.04%
		% of chrY on-target	96%	94%	100%	99%	98%	96%
	Condition 1	Condition 2						
Fold-enrichment (down-sampled data)	YCC	Pre-capture	7039.6	9549.0	17.0	27.0	250.0	12.0
	YCC	WGC	963.8	1273.2	170.0	270.0	25.0	12.0
	YCC	WGC + YCC	3.8	8.6	1.0	0.3	0.2	0.5
	WGC	Pre-capture	7.3	7.5	0.1	0.1	0.0	0.0
	WGC + YCC	Pre-capture	1843.3	1110.0	17.1	81.3	1097.0	24.5
	WGC + YCC	WGC	252.4	148.0	171.0	813.0	109.7	24.5

Ten replicates per library were obtained by down-sampling to the minimum number of retained reads within each sample (underlined in “Total data” rows). The “Mapping to chrY” section indicates the number of unique reads mapping to the Y-chromosome. “On-target” refers to the unique on-target reads. “%of sequenced on-target” and “% of chrY on-target” refer to the percentage of on-target reads with respect to the total sequenced reads and to the total reads mapping to the Y-chromosome, respectively. The fold-enrichments were calculated with the down-sampled data, by dividing the number of on-target reads in Condition 1 by the number of on-target reads in Condition 2; when the denominator was 0, we assigned the number of on-target reads in Condition 1

To calculate the enrichment rate, we used the subsampled data and computed the average number of unique reads mapped to the on-target regions in each experiment. Then, we calculated fold-enrichment by dividing the on-target average of YCC or WGC + YCC experiments by that of the pre-capture libraries. Specifically, we calculated this fold-enrichment for YCC using the pre-capture condition as a baseline and compared the WGC + YCC experiments to both the pre-capture and WGC conditions. For the cases with replicates with no reads aligning to the Y chromosome or target regions, we used the maximum number of reads observed across the replicates of a given library as a baseline. We computed binomial proportion confidence intervals for the mean endogenous content, the proportion of on- and off-target reads, and clonality, and we conducted a *t* test for the length estimate. All statistical tests were computed in R software, version 3.3.1 [37].

To call Y-chromosome genotypes for each sample, we first merged data across experiments. We used the haploid genotype caller implemented in ANGSD, retaining only bases with quality scores of at least 13 and sampling one random base at each site [38]. Finally, we performed a binary tree search with a custom script to find the most derived SNP that determines the haplogroup of the individuals. We used as input the phylogenetic tree constructed from the Y-SNPs reported in Phase 3 of the 1000 Genomes Project [4].

### Sex determination

To determine the biological sex of the six individuals, we used the script in [39] to calculate from the ratio (*R*_y_) of reads mapping to the Y-chromosome to those mapping to both sex chromosomes [39]. *R*_y_ values above 0.075 are consistent with a male genotype [39].

### Yield and enrichment curves

We estimated the yields and complexities of the libraries, with respect to the reads mapping to the targeted regions, with the PreSeq package implemented in R (preseqR, [40]) and corrected the amount of required sequencing by the fraction of on-target reads in the libraries. Since the method relies on having a fraction of duplicated reads to estimate the yield, for the cases where the pre-capture libraries did not have duplicated on-target reads to adjust a yield curve, we instead assumed a linear relationship with a slope equal to the proportion of unique on-target reads present in the library. We then modeled an “enrichment curve” to explore the level of enrichment predicted for different amounts of sequencing. To this end, we used the median unique on-target reads as returned by PreSeq to calculate an expected fold-enrichment. We divided the median value estimated by PreSeq of each captured library by the median of its pre-captured counterpart (i.e., YCC vs. pre-capture, WGC + YCC vs. pre-capture, and WGC + YCC vs. WGC).

## Results

### Enrichment rates

**Fig. 1:**
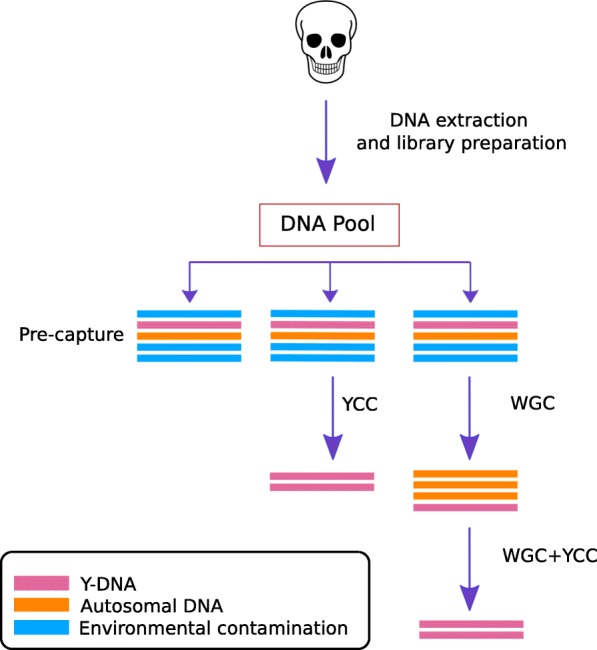
Experimental enrichment scheme. We have four different conditions: Pre-capture, YCC, WGC, and WGC + YCC. The pre-capture condition is our initial library preparation prior to any enrichment. The WGC is designed to target all autosomal and sex chromosomes. The Y-capture in the YCC and the WGC + YCC conditions targets ~ 10.3 Mb of Y-chromosome sequence

We tested the performance of Y-chromosome capture on Illumina sequencing libraries obtained from the archaeological remains of six individuals excavated in the Caribbean islands of Saint Martin (STM1 and STM2) and Puerto Rico (PI174, PI383, PI435, and PI437) (Table 1). For each sample, we performed a series of enrichment experiments, as depicted in Fig. 1. First, we shotgun-sequenced a DNA library without performing any enrichment. We then performed a capture reaction targeting a set of DNA probes covering ~ 8.9 Mb of the non-recombining portion of the Y chromosome*.* These regions were validated by Poznik and colleagues in [2] as being well suited for unambiguous read mapping and for yielding reliable genotype and haplogroup calls from short-read sequencing. Additionally, we performed another set of capture experiments, either enriching only the whole-genome (WGC) or the on-target regions after having enriched the whole genome (WGC + YCC). We confirmed the molecular sex of the samples and determined that the six individuals each had a karyotype consistent with XY [39]. We then assessed the performance of the capture experiments.

**Fig. 2:**
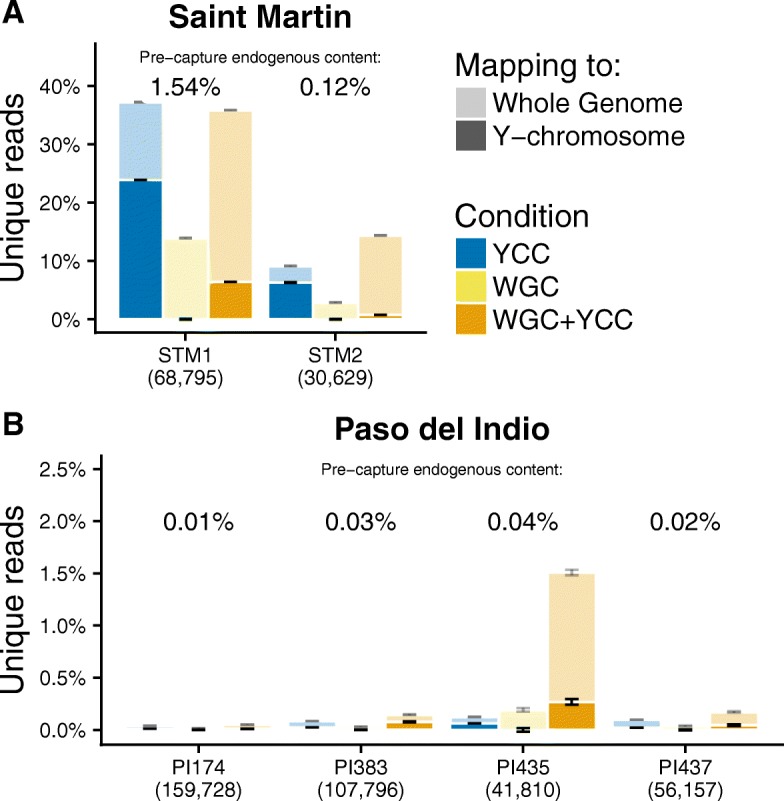
Endogenous DNA content in enriched libraries. Percentage of the unique retained reads that aligned to the human genome in (**a**) Saint Martin and (**b**) Puerto Rico samples. “STM” stands for Saint Martin and “PI” for Paso del Indio, Puerto Rico. The percentages in parentheses below the *x*-axis indicate the number of down-sampled reads per library. The error bars represent 95% confidence intervals of endogenous DNA content found in the samples across the 10 down-sampled replicates. Darker colors correspond to the proportion of the unique reads that aligned to the Y chromosome. Whole-genome enriched libraries have < 0.04% reads aligning to the Y chromosome

Since experiments yielded differing numbers of reads per sample, we down-sampled to equal numbers per individual as described in Methods (Table 1). The pre-capture libraries yielded 0.01 to 1.54% unique reads aligning to the human genome (Fig. 2), and less than 0.004% mapping to the Y-chromosome. After implementing the YCC and WGC + YCC enrichments, the endogenous DNA content increased, on average, by factors of 24.2 to 122.0 for the STM samples (Fig. 2a) and by factors between 3.2 and 38.9 for the PI samples (Fig. 2b). Moreover, in the YCC and WGC + YCC libraries, 5.3 to 68.3% of the human STM reads mapped to the Y chromosome, with 17.7 to 50.0% the corresponding figures for the PI samples.

**Fig. 3:**
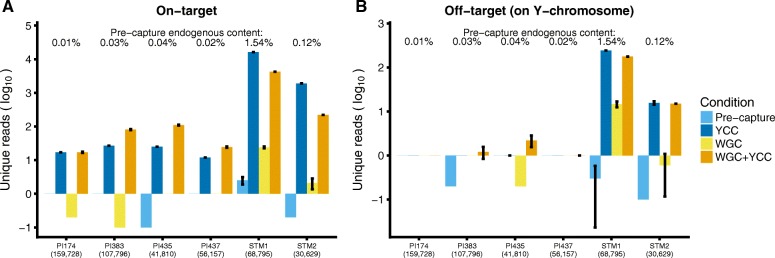
On- and off-target reads. We show the mean and the 95% confidence interval for 10 replicates. **a** Unique reads mapping to the Y-chromosome target regions. **b** Unique reads mapping to the Y chromosome but not to the targeted regions. For some libraries, no reads mapped to the Y chromosome, across the 10 replicates

**Fig. 4:**
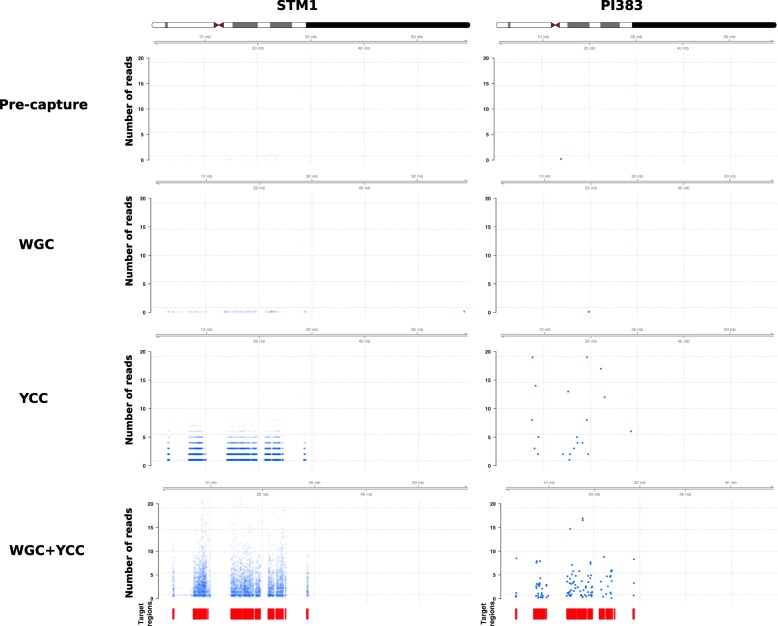
Depth of coverage across the Y-chromosome. From top to bottom, rows depict the coverage levels for the pre-capture, YCC, WGC and WGC + YCC conditions. Red boxes represent the targeted regions. Each blue point represents sequencing coverage within a 1000-bp window, averaged across 10 subsampled replicates per sample per condition explaining depths of coverage below 1. To help with readability, we increased the opacity of the points in the PI383 column

To evaluate whether the enrichment experiments effectively recovered the targeted regions, we compared the total number of unique reads mapping to the targeted regions as well as the number of off-target reads (Fig. 3). YCC experiments on the STM samples yielded 7039 to 9549-fold increases of on-target sequences compared to the pre-capture condition (Table 1). The WGC + YCC experiments on the same samples resulted in 148- to 252.35-fold-enrichment compared to WGC alone. For the PI samples, YCC experiments resulted in 12- to 250-fold enrichment, and we observed 24.5- to 813-fold enrichment for the WGC + YCC approach. Although we observed an increase in the off-target content for the STM enrichments (Fig. 3), it is one order of magnitude smaller than their respective on-target enrichment. Above 94% of all reads mapping to the Y chromosome were on target in both YCC and WGC + YCC, in contrast to the pre-capture and WGC experiments where these figures range between 25 and 75% (Table 1, Total data section). Overall, the distribution of the on-target sequences in all Y-chromosome enrichment experiments is qualitatively even (Fig. 4, Additional file 2: Figure S1). In summary, all Y-chromosome capture enrichment experiments consistently increased the number of unique on-target reads.

### Length distribution and clonality

**Fig. 5:**
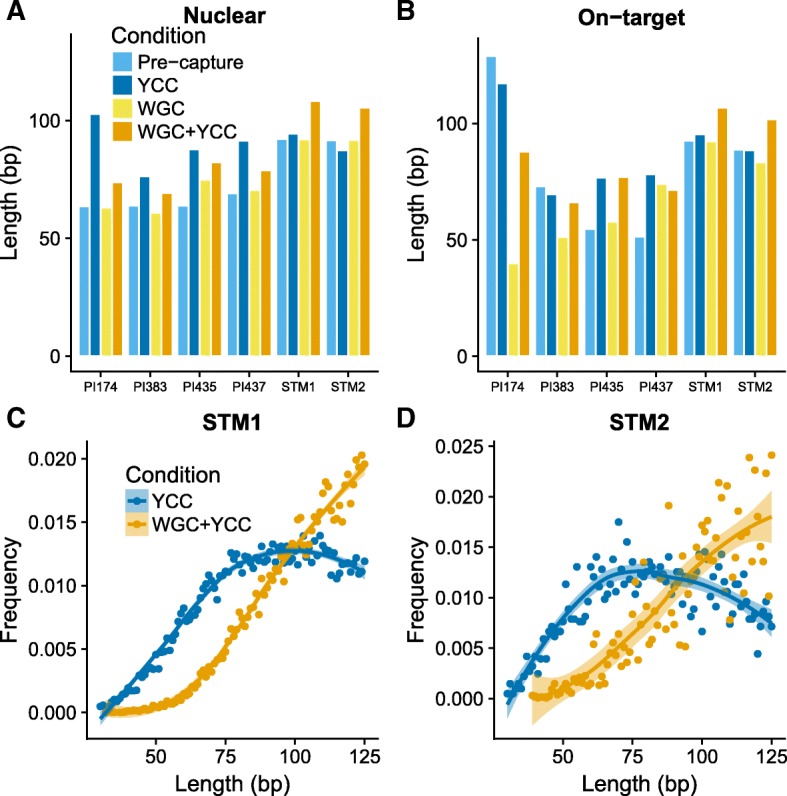
Lengths of mapped reads. **a** Reads aligned to the nuclear genome. **b** On-target reads. **c** and (**d**) depict the length distributions of reads mapping to the whole genome for STM1 and STM2 samples, respectively. The length distribution was smoothed by fitting a polynomial curve to the observed frequencies; the ribbons correspond to 95% confidence intervals

**Fig. 6:**
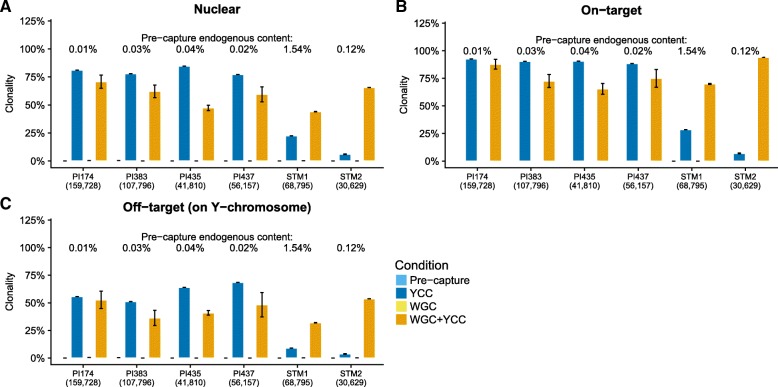
Clonality. **a** Clonal reads mapping to the nuclear human genome. **b** Clonality of the reads mapping to the targeted regions. **c** Clonal reads mapping to the Y chromosome but not to the targeted regions. Error bars represent 95% confidence intervals across 10 subsampled replicates

To explore which features of the pre-capture libraries may have influenced the differences in enrichment rates between the STM and PI groups, we contrasted the lengths and complexities of the individual libraries across experiments. The extraction protocols differ between the STM and the PI samples (see Table 1). This should impact the read length distribution and we therefore avoided to compare absolute values across samples and instead performed paired tests. We observed a significant trend toward longer fragments after enrichment for all experiments (Fig. 5, Additional file 3: Figure S2) (paired samples *t-test*, *p*-value = 0.002), consistent with previous findings [8, 17, 41]. Reads from the two STM samples were 91.7 to 92.1 base pairs (bp) long for the pre-capture condition, 87.4 to 94.5 bp after YCC, and 105.5 to 108.3 bp long after WGC + YCC. Likewise, whereas the average length of PI reads in the pre-capture libraries ranged from 63.6 to 69.1 bp, average lengths increased to 76.3–102.3 and 69.3–82.2 bp after YCC (paired samples *t-test*, p-value = 0.02) and WGC + YCC (paired samples *t-test*, p-value = 0.03), respectively. On-target clonality levels (percentage of PCR duplicates) were considerable for all samples in the YCC and WGC + YCC experiments. We observed 9.2 to 28.6% clonality for YCC of the STM samples at 68,795 and 30,629 down-sampled reads. The remaining WGC + YCC (STM), and all WGC + YCC and YCC (PI) libraries had greater clonality values, ranging from 65.4 to 94.1% (Fig. 6). We did not observe on-target duplicates with which to calculate the clonality in any of the down-sampled pre-capture libraries (Additional file 4: Table S2), so instead we used the whole data to estimate the clonality. For STM1, the pre-capture library had 10.0% on-target clonal reads (for 34,025,874 sequenced reads, Additional file 4: Table S1), while for all the other pre-capture libraries the on-target clonality is 0%, as they have 1 to 197 reads on-target (Additional file 4: Table S1).

### On-target yield for aDNA libraries

**Fig. 7:**
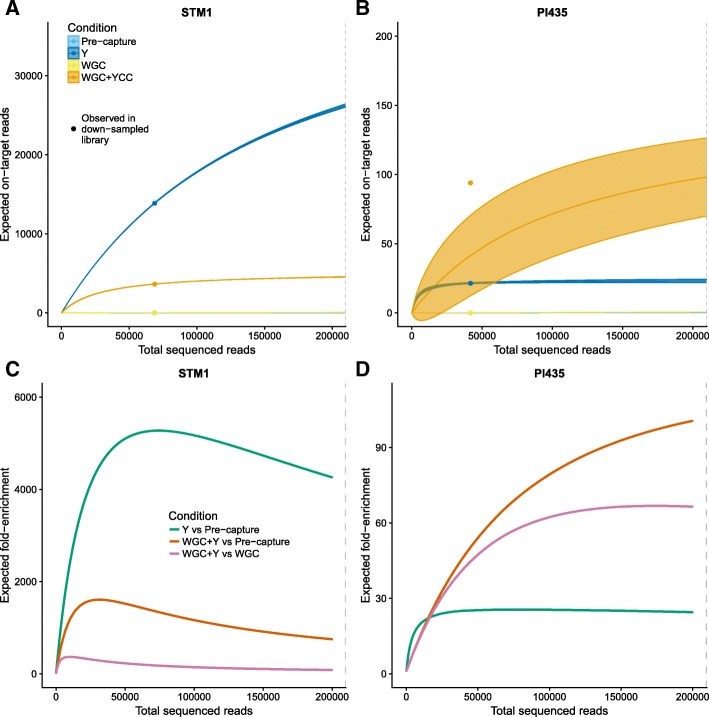
Expected yield and on-target fold-enrichment. Dashed lines indicate the number of down-sampled reads. **a** and (**b**): Predicted median value and variance (across 100 bootstrap replicates) of the number of on-target reads, as a function of total sequenced reads. The points depict the observed numbers of on-target reads in the down-sampled libraries. **c** and (**d**): Expected enrichment of on-target reads versus number of sequenced reads for each condition and each sample

The yield curves for both capture conditions (YCC and WGC + YCC) corroborate the high clonality of the PI libraries (Fig. 7a, b). We observed that the YCC libraries of these samples plateaued at very shallow sequencing, saturating at ~ 25,000 sequenced reads, compared to saturations at 50,000–100,000 sequenced reads for the WGC + YCC libraries. However, the complexity curves indicate that after sequencing 100,000 reads of the WGC + YCC libraries, we would not retrieve more than 150 different reads, regardless of the capture approach. On the other hand, although we sequenced fewer than 100,000 reads for each of the YCC experiments on the STM individuals, the complexity curves suggest that these libraries could be further sequenced to increase the coverage of the targeted regions.

### Enrichment curves based on yield estimates

We computed enrichment curves (Fig. 7c, d) to predict enrichment rates for deeper sequencing. Consistently, we observe that the YCC of STM2 (Additional file 5: Figure S3 H) recovers 1790 more on-target reads than the pre-capture experiment at the down-sample point. However, as it has not reached saturation, this enrichment value can increase to at least 5000-fold, similar to the maximum enrichment of STM1 (Fig. 7a). In contrast, the projected enrichment of the PI WGC + YCC libraries at 200,000 total reads is only 100- and 72-fold versus the pre-capture and WGC libraries, respectively. We also note that, for PI samples, the three enrichment rates calculated “decelerate” at 100,000 sequenced reads or fewer, once more reflecting the low complexity levels of the initial pool (Fig. 7, Additional file 5: Figure S3).

### Y haplogroup calls

Finally, we combined all the sequence data generated for each sample to call Y-chromosome haplogroups. With the new data generated, we were able to call haplogroups for both STM individuals. The combined on-target depth of coverage was 0.04× to 0.28×, with a sequencing depth of at least one for 0.32–1.84 Mb (Table 2, Additional file 6: Figure S4 for Y-SNPs coverage). In the STM1 individual we observed derived alleles belonging to the R1b-M343 clade, consistent with its previously reported haplogroup (R1b1c-V88) [24]. In the same study, even after WGC, the haplogroup for STM2 could not be resolved [24]. Only after integrating the previously generated data with that produced in this study, were we able to assign the Y-haplogroup as E1b1a1a1-M80. Regarding the PI samples, we did not find any reads covering the SNPs in the database in the pre-capture or the WGC libraries. After YCC or WGC + YCC, we observe between 5 and 74 variant sites per library, leading to the identification of haplogroups defining broader regions. For instance, the haplogroup found for PI383, P-M45, is the immediate ancestor of haplogroups R and Q. In Table 2, we show the haplogroups for all the individuals according to the most derived SNP identified in each condition.

**Table 2:** Numbers of Y-chromosome bases, Y-SNPs and haplogroups retrieved

Sample	Condition	Positions recovered	Total SNPs	Ancestral SNPs	Derived SNPs	Haplogroup retrieved
STM1	All	2,205,331	12,061	11,625	436	R1b-M343
	Pre-capture	93,725	595	577	18	R1b-M343
	YCC	1,372,731	8103	7805	298	R1b-M343
	WGC	354,255	2414	2321	93	R1b-M343
	WGC + YCC	493,085	2971	2871	100	R1b-M343
STM2	All	428,846	2183	2091	92	E1b1a1a1-M80
	Pre-capture	19,994	109	100	9	E1b1a1a1-M80
	YCC	220,732	1262	1215	47	E1b1a1-M2
	WGC	109,411	760	726	34	E1b1a1a1-M80
	WGC + YCC	219,152	114	110	4	CT-M168
PI174	All	3224	19	18	1	A1-V168
	Pre-capture	129	0	0	0	–
	YCC	1993	12	11	1	A1-V168
	WGC	147	2	2	0	A1-V168
	WGC + YCC	1818	11	10	1	A1-V168
PI383	All	7738	46	45	1	P-M45
	Pre-capture	146	0	0	0	–
	YCC	1809	13	12	1	P-M45
	WGC	473	5	5	0	P-M45
	WGC + YCC	6890	42	41	1	P-M45
PI435	All	16,469	100	97	3	BT-M42
	Pre-capture	164	0	0	0	–
	YCC	1918	15	15	0	BT-M42
	WGC	2632	18	18	0	–
	WGC + YCC	12,399	86	83	3	BT-M42
PI437	All	3444	14	14	0	–
	Pre-capture	103	0	0	0	–
	YCC	938	6	6	0	–
	WGC	296	0	0	0	–
	WGC + YCC	2320	11	11	0	–

## Discussion

In this paper, we have described the efficiency of Y-DNA recovery from in-solution Y-chromosome capture-enrichment experiments and from different WGC protocols followed by Y-chromosome enrichment on aDNA libraries obtained from the archaeological remains of six males excavated in the Caribbean and dating between 300 and 2000 years old. We performed silica-based extraction and built double stranded libraries for all samples (see Table 1). The experimental design involved the targeted enrichment of 8.9 Mb of the Y chromosome on both standard and WGC libraries. As the WGC enrichment protocols differed between sample groups, we evaluated the success of the enrichment using within-sample comparisons. Overall, both approaches succeed in increasing the proportion of on-target sequences as compared to pre-capture libraries. For both enrichment approaches and for all samples, we observed that most of the sequenced reads mapped to the targeted regions. Moreover, we succeeded in assigning refined haplogroups by sequencing between 40,000 and 70,000 reads of Y-chromosome captured libraries for the two samples with endogenous content above 0.1% (0.12 and 1.54%, respectively). Finally, despite having successfully increased the yield of Y-chromosome reads in every instance we tested, our results suggest that the amount of data retrieved with this capture strategy will only be meaningful (i.e. enough to call a haplogroup or to inform a Y-phylogeny) if the starting libraries have adequate complexity and endogenous content. Note that further work on samples with more variables endogenous contents and clonality is needed to make quantitative statements regarding the complexity levels and the endogenous amounts that are prohibitive for cost-effective capture experiments. Moreover, it would be interesting to compare the effect of the extraction methods on the efficiency of the capture experiments.

### Factors influencing enrichment

We observed consistent enrichment of Y-DNA on aDNA libraries. However, we also observed marked differences of the performance between samples (STM and PI) and library types (standard vs. WGC libraries). We tentatively conclude that this is due to differences in starting endogenous content, read-length distributions, and the complexities of the libraries. Although it is now possible to increase the endogenous content of poorly preserved tissues, it has indeed been shown that in-solution capture enrichment techniques perform better on samples with starting endogenous content greater than 1% and with little clonality [17]. Although we analyzed only six samples, it is worth noting that our results are consistent with this previously reported threshold. Indeed, as expected, the PI sample enrichment levels were systematically lower than those in the STM samples. Low complexity levels in the starting libraries also hamper the success of capture experiments, as these protocols usually involve an amplification step, which further increases the clonality. In addition, the enriched libraries were subject to an increase in the fragment length most likely driven by the probe length (105 bp) (Fig. 5). However, the shift was more pronounced for the PI samples, suggesting that a substantial proportion of the shorter fragments in the pre-capture and WGC libraries was not retrieved in the YCC and WGC + YCC. Together, these observations provide useful insights as to the features in standard libraries that should be considered when planning capture experiments, thereby opening avenues for investigating ways to optimize these protocols.

Subsequently, we noted that for the STM samples the YCC experiments yielded higher fold-enrichments of the targeted regions than did the WGC + YCC captures. In contrast, for the PI samples, the WGC + YCC performed better. While we believe the difference owes mostly to the endogenous content, it could also be in part due to the techniques employed to enrich the whole genomes of the STM and PI samples (MYbaits and WISC, respectively). Indeed, although based on the same molecular principle, those technique have slight differences in their performances [17]. However, despite the slightly higher enrichment rates in WGC + YCC libraries, as compared to YCC, drawing from our results, we do not recommend implementing enrichments for libraries similar to the PI libraries. As we show here, despite having used a more efficient extraction protocols for those samples with low endogenous content (Table 1), regardless of the capture approach and the total sequenced reads, enriched PI libraries recover only a few hundred of Y-chromosome reads. Consequently, the ability to identify informative SNPs for haplogroup assignment is limited in these cases.

### Implications of Y-haplogroup assignments for the samples

Finally, the haplogroup inference was effective only for the two STM samples, for which we recovered at least 0.32 Mb of the Y-chromosome (3.7% of on-target regions). The STM1 individual bore the M343 mutation characteristic of haplogroup R1b, consistent with the back-to-Africa R1b1c-V88 haplogroup reported in [24]; however, we did not observe any of the SNPs specific to the V88 branch. Whereas for STM2, we identified a common and widespread African haplogroup characteristic of the Bantu expansion [42], E1b1a1a1-M80, consistent with the results from the analyses of the autosomal chromosomes [24]. For the remaining individuals, we could not resolve haplogroups due to the reduced complexity and endogenous content of the libraries. Additionally, our results might be impacted by the paucity of Y-SNPs that define the tips of the Native American haplogroups versus haplogroups from other well-characterized populations in the database employed. For example, no Puerto Rican males in the 1000 Genomes dataset bore Native American Y haplogroups. Rather, all possessed European or African lineages, primarily belonging to R1b and E1b clades [4], reflecting sex-biased admixture patterns during European colonization of the island (and reproduced across the Americas) [43]. This fact highlights the need to assay ancient genetic variation among pre-contact Native American samples. It is often challenging to recover DNA for such samples and the enrichment method we discuss here would certainly help in those cases.

## Conclusions

In the past decade, new technologies and protocol improvements have emerged to efficiently recover ancient DNA. However, the endogenous DNA fraction continues to be a limiting step in ancient genomics studies. The first efforts to overcome this limitation have focused on targeting the mtDNA, because it is relatively short (~ 16 kB), and it is present in multiple copies per cell, unlike the autosomes (two copies) and the Y chromosome (one copy). For the Y chromosome, targeted enrichment strategies are more problematic due to its richness in repetitive and palindromic sequences. For the same reasons, Y-chromosome content is relatively poor in WGC studies, although WGC is becoming a cost-effective alternative for ancient genomics. Therefore, we used previously reported high-quality regions to capture the most phylogenetically informative portion of the Y chromosome. We confirmed the effectiveness of the method by noting that, after capture, up to 99.1% of the reads mapping to the Y chromosome fall within the targeted regions. In this study, the two libraries with endogenous DNA content of 0.12 and 1.54% yielded ~ 10-fold greater enrichment rates under YCC, as compared to WGC + YCC libraries. Despite observing a greater enrichment for WGC + YCC experiments in the four samples with low endogenous DNA proportions (0.01 to 0.04%), none of the enriched libraries yielded enough Y-chromosome SNPs to assign a haplogroup. Moreover, we observed that both YCC and WGC + YCC libraries outperformed pre-capture libraries with respect to Y-DNA content, the data generated in this study do not allow us to assert yet if carrying out WGC is advantageous or not before enriching for Y-DNA capture. However, as could be expected based on previous work [17, 44], initial levels of endogenous DNA content, library complexities and fragment lengths of the starting libraries seem to influence the performance of the libraries after consecutive rounds of capture experiments. We thus stress the need to consider the initial complexity, endogenous DNA content, and read lengths when planning these experiments. We recommend a design that includes the estimation of predictive yield and enrichment curves [40, 45], based on shallow sequencing, to inform the best sequencing strategy and avoid sequencing beyond saturation.

Finally, there is a vast potential to incorporate Y-chromosome information from aDNA samples into the study of human population history from regions beyond Eurasia. In our work, we go beyond SNP capture and present the first instance of Y-chromosome capture on ancient samples, opening new avenues of research to improve the performance of these experiments and to extract Y-chromosome information from ancient samples.

## Additional files

Additional file 1: On-target regions. (BED 90 kb)

Additional file 2: **Figure S1.** Depth of coverage across the Y-chromosome. From top to bottom, rows depict the coverage levels for the pre-capture, YCC, WGC and WGC + YCC conditions. Red boxes represent the targeted regions. Each blue point represents sequencing coverage within a 1000-bp window, averaged across 10 subsampled replicates per sample per condition, explaining depths of coverage below 1. To improve readability, we increased the opacity of the points in the PI samples. (PDF 1474 kb)

Additional file 3: **Figure S2.** Length distribution of mapped reads. Length distributions of reads mapping to the whole genome. The length distribution was smoothed by fitting a polynomial curve to the observed frequencies; the ribbons correspond to 95% confidence intervals. (PDF 45 kb)

Additional file 4: **Table S1.** Summary of sequenced and mapped reads of the complete dataset. **Table S2.** Mean and standard errors regarding reads from down-sampled libraries. (XLSX 25 kb)

Additional file 5: **Figure S3.** Expected yield and on-target fold-enrichment. Dashed lines indicate the number of down-sampled reads. (A-F): Predicted median value and variance (across 100 bootstrap replicates) of the number of on-target reads, as a function of total sequenced reads. The points depict the observed numbers of on-target reads in the down-sampled libraries. (G-L): Expected enrichment of on-target reads versus number of sequenced reads for each condition and each sample. (PDF 245 kb)

Additional file 6: **Figure S4.** Average numbers of Y-SNPs covered at least once. For a certain depth of coverage (x-axis), the dots represent the average number of SNPs (y-axis) observed in the ten replicates. The bars represent the standard error. (PDF 9 kb)

## Abbreviations

WGC + YCC: Whole Genome Capture + Y-chromosome capture
WGC: Whole genome capture
YCC: Y-chromosome capture
