# Supplementary figures and images for: In-solution Y-chromosome capture-enrichment on ancient DNA libraries
Source: BMC Genomics. 2018 Aug 14;19:608. doi: 10.1186/s12864-018-4945-x (PMC6092841; doi:10.1186/s12864-018-4945-x)

**STM1**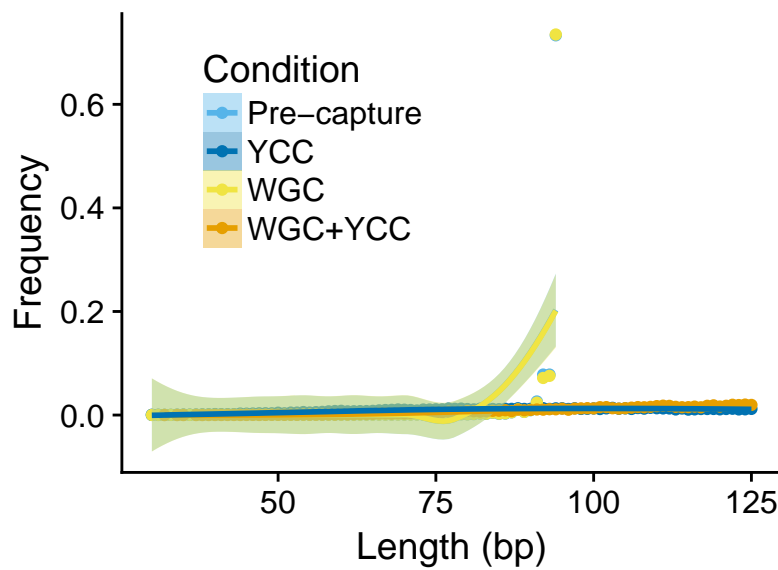**STM2**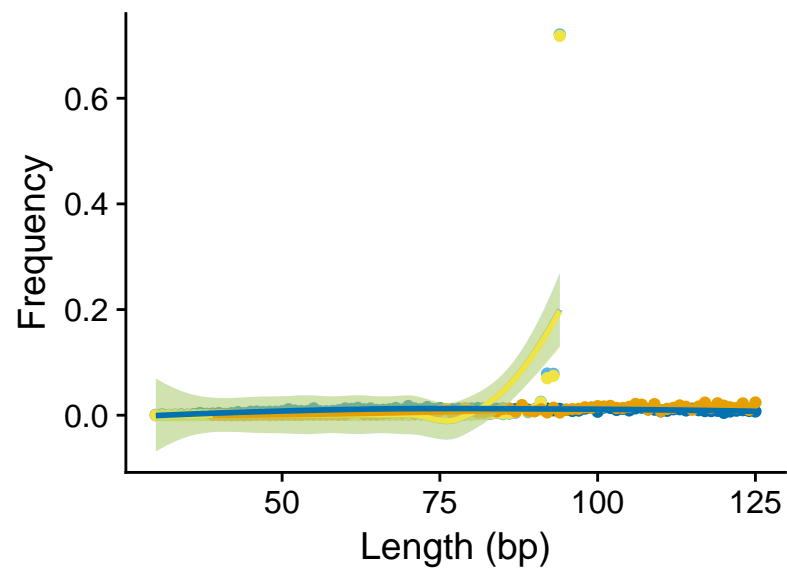**PI174**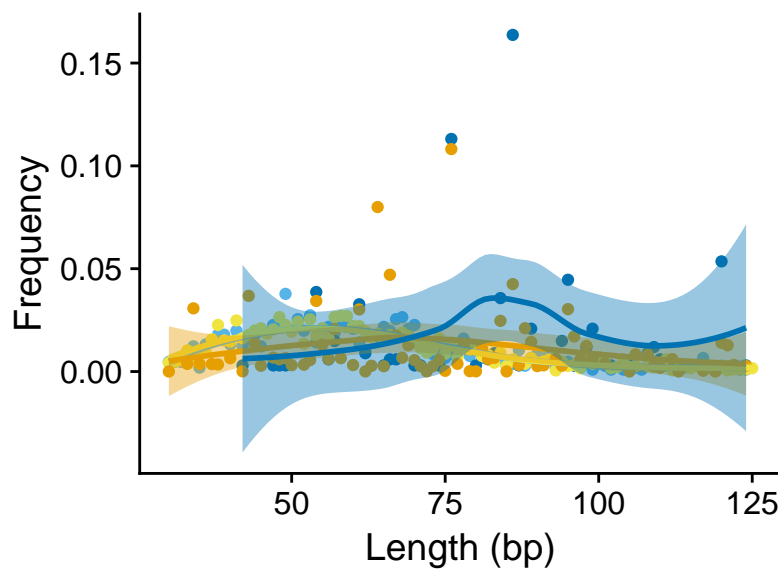**PI383**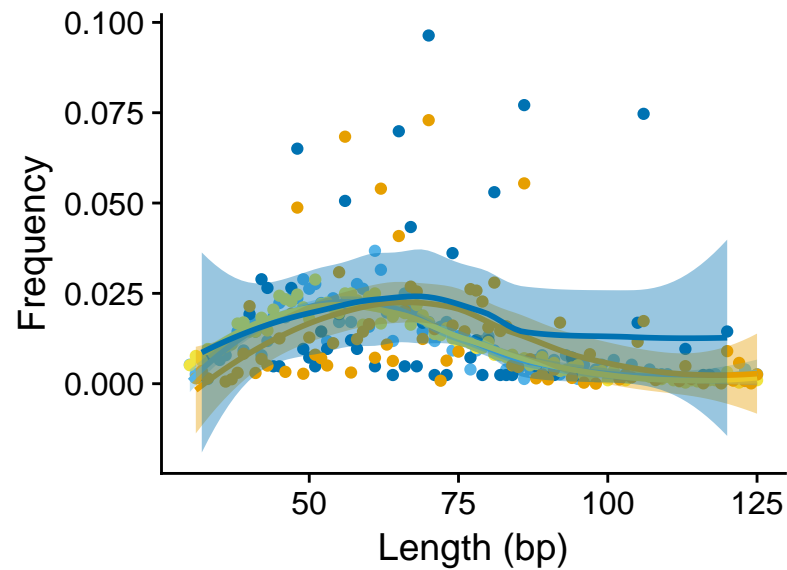**PI435**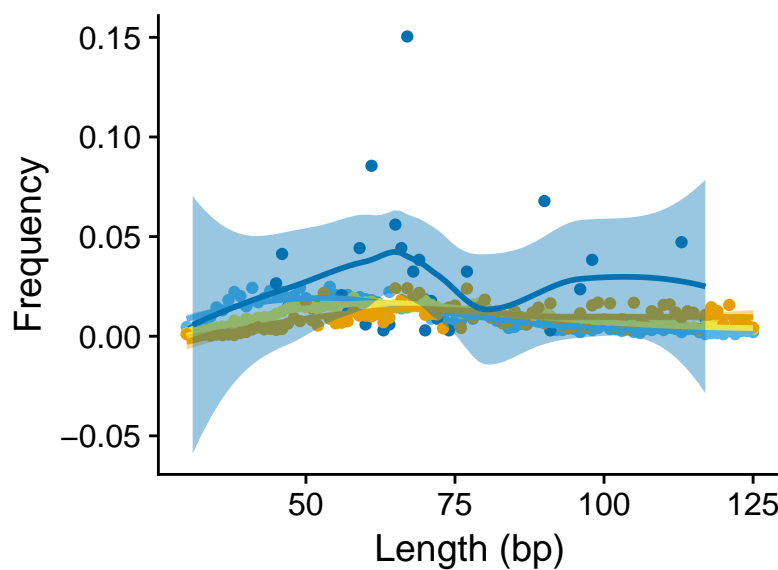**PI437**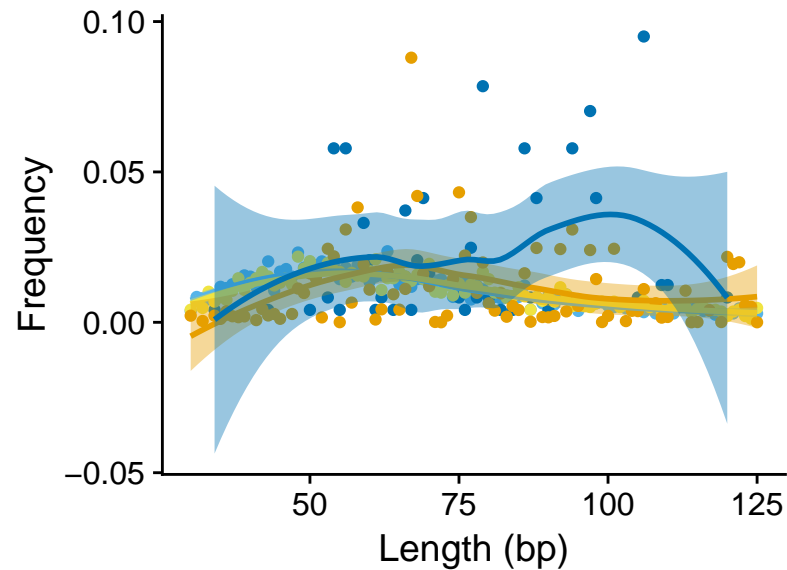

Supplement: Supplementary file 3 — Figure S2. Length distribution of mapped reads. Length distributions of reads mapping to the whole genome. The length distribution was smoothed by fitting a polynomial curve to the observed frequencies; the ribbons correspond to 95% confidence intervals. (PDF 45 kb) [file 12864_2018_4945_MOESM3_ESM.pdf]

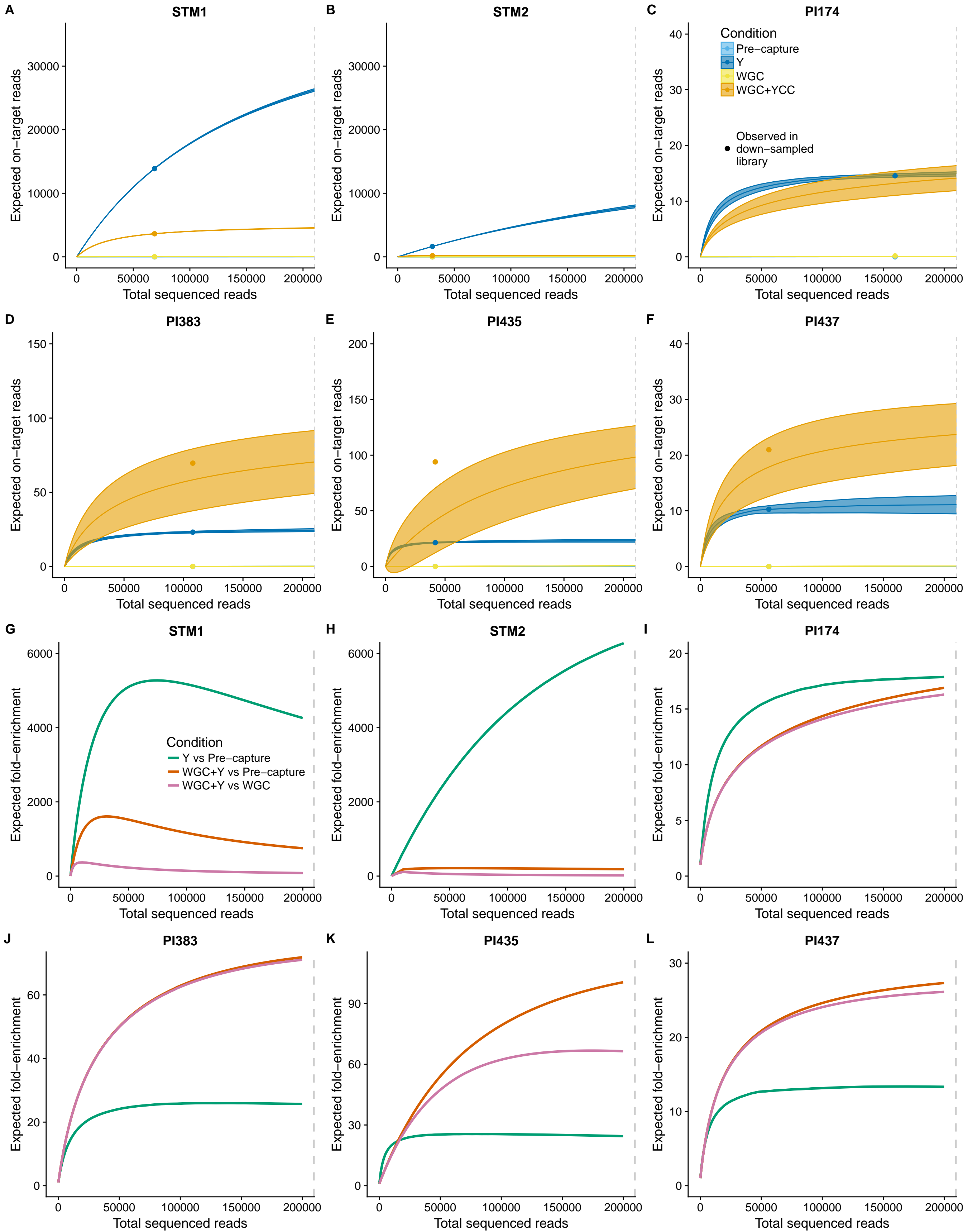

Supplement: Supplementary file 5 — Figure S3. Expected yield and on-target fold-enrichment. Dashed lines indicate the number of down-sampled reads. (A-F): Predicted median value and variance (across 100 bootstrap replicates) of the number of on-target reads, as a function of total sequenced reads. The points depict the observed numbers of on-target reads in the down-sampled libraries. (G-L): Expected enrichment of on-target reads versus number of sequenced reads for each condition and each sample. (PDF 245 kb) [file 12864_2018_4945_MOESM5_ESM.pdf]

**STM1**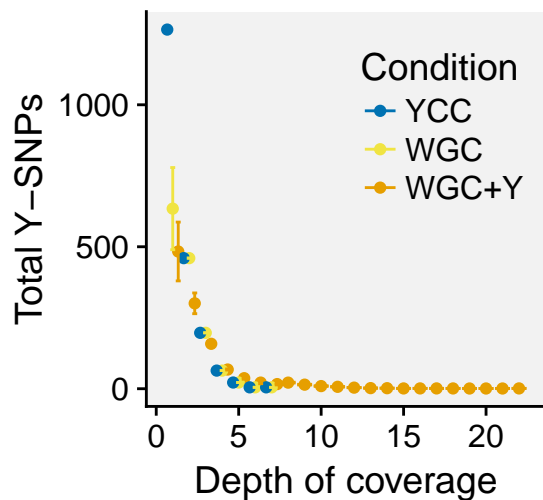**STM2**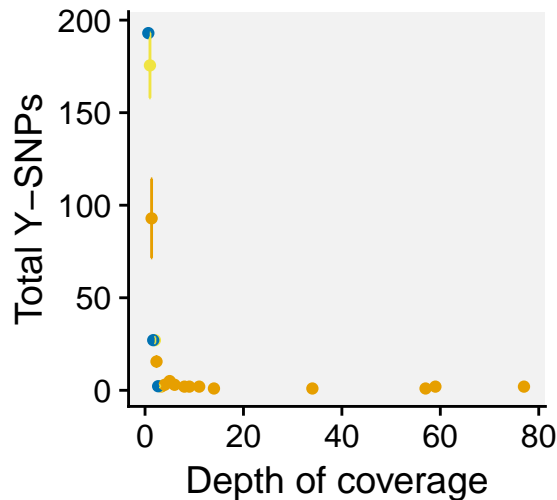**PI174**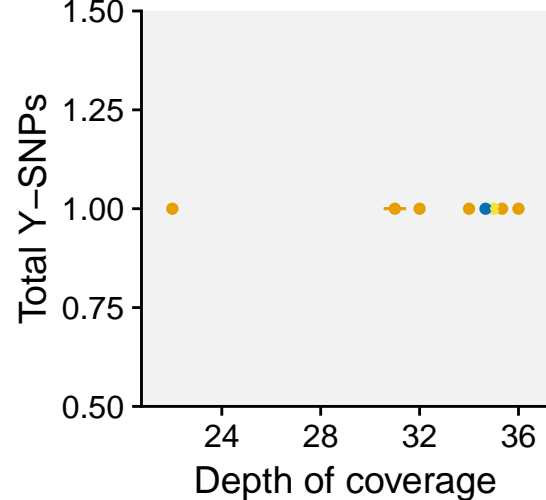**PI383**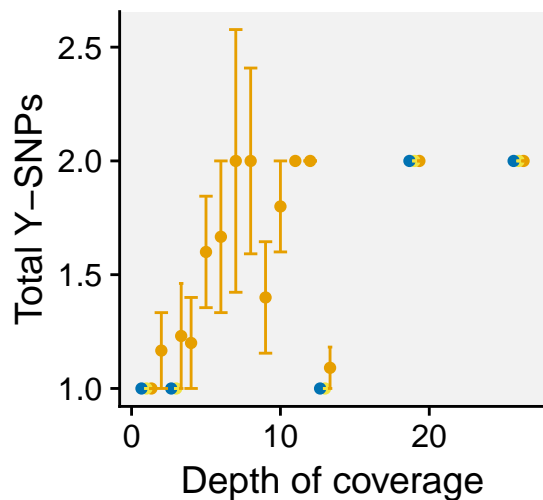**PI435**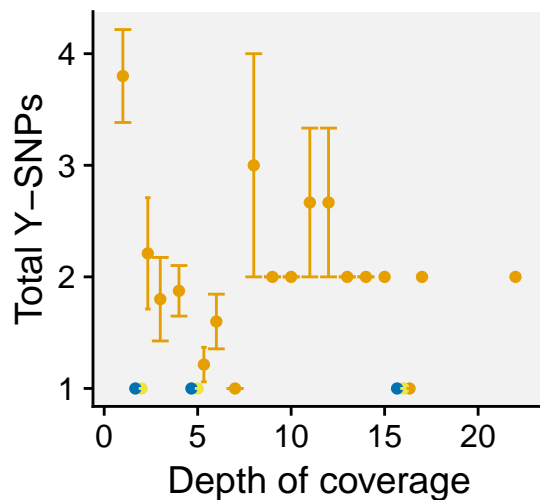**PI437**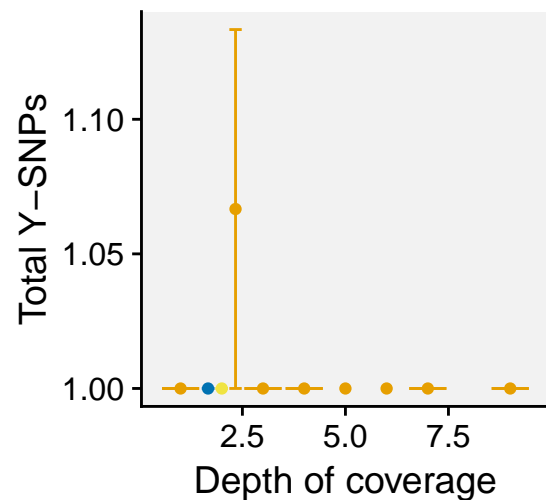

Supplement: Supplementary file 6 — Figure S4. Average numbers of Y-SNPs covered at least once. For a certain depth of coverage (x-axis), the dots represent the average number of SNPs (y-axis) observed in the ten replicates. The bars represent the standard error. (PDF 9 kb) [file 12864_2018_4945_MOESM6_ESM.pdf]
