# Supplementary material for: In-solution Y-chromosome capture-enrichment on ancient DNA libraries
Source: BMC Genomics. 2018 Aug 14;19:608. doi: 10.1186/s12864-018-4945-x (PMC6092841; doi:10.1186/s12864-018-4945-x)
Supplement: Supplementary file 2 — Figure S1. Depth of coverage across the Y-chromosome. From top to bottom, rows depict the coverage levels for the pre-capture, YCC, WGC and WGC + YCC conditions. Red boxes represent the targeted regions. Each blue point represents sequencing coverage within a 1000-bp window, averaged across 10 subsampled replicates per sample per condition, explaining depths of coverage below 1. To improve readability, we increased the opacity of the points in the PI samples. (PDF 1474 kb) [file 12864_2018_4945_MOESM2_ESM.pdf]

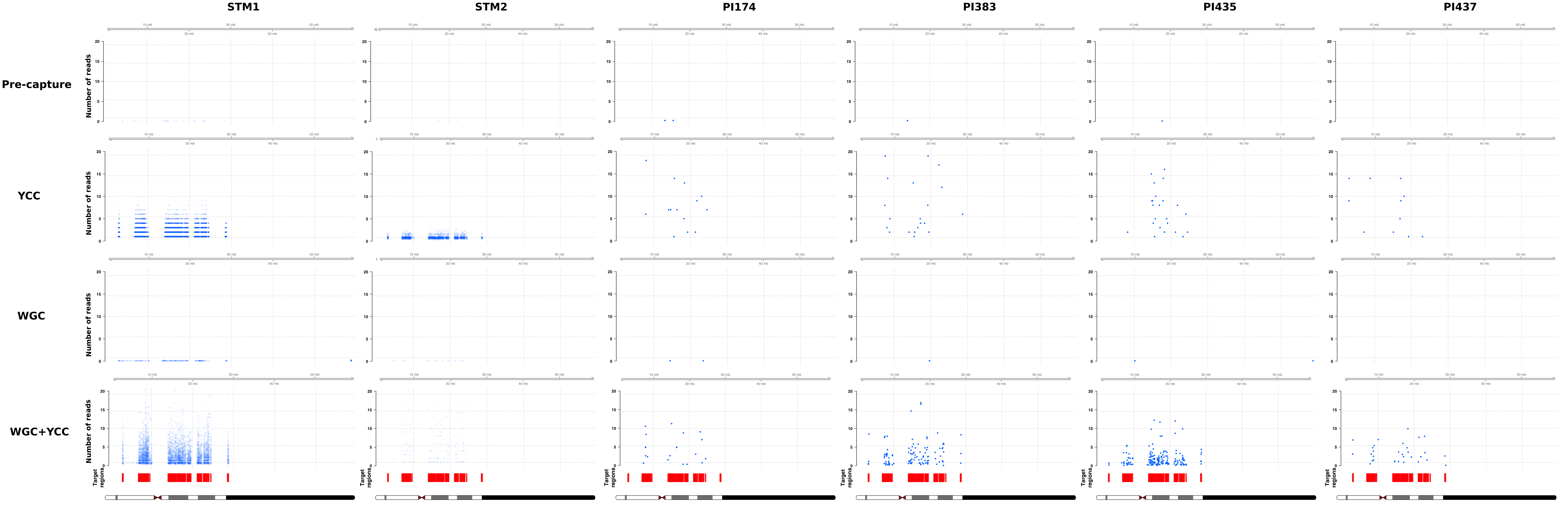

STM1
STM2
PI174
PI383
PI435
PI437
Pre-capture
YCC
WGC
WGC+YCC
Number of reads
Target regions
10 mb
20 mb
30 mb
40 mb
50 mb
0
5
10
15
20
